# Is All Fat Created Equal? A Comparative Study of Chondrogenesis Potential of Peri-Ovarian Adipose Tissues in Dogs

**DOI:** 10.3390/ani16121900

**Published:** 2026-06-19

**Authors:** Mirko Sergio, Giorgio Mirra, Riccardo Giorgino, Anna Lange-Consiglio, Valeria Martini, Silvia Clotilde Modina, Liliana Carnevale, Maria Cristina Veronesi, Chiara Bazzocchi, Paola Pocar, Chiara Stocchero, Barbara Canciani, Valentina Rafaela Herrera Millar, Alessia Di Giancamillo

**Affiliations:** 1 Department of Veterinary Medicine and Animal Sciences, University of Milan, 26900 Lodi, Italy; mirko.sergio@unimi.it (M.S.); anna.langeconsiglio@unimi.it (A.L.-C.); valeria.martini@unimi.it (V.M.); silvia.modina@unimi.it (S.C.M.); liliana.carnevale@unimi.it (L.C.); maria.veronesi@unimi.it (M.C.V.); chiara.bazzocchi@unimi.it (C.B.); paola.pocar@unimi.it (P.P.); chiara.stocchero@unimi.it (C.S.); 2 Department of Biomedical Sciences for Health, University of Milan, 20133 Milan, Italy; giorgio.mirra@unimi.it (G.M.); alessia.digiancamillo@unimi.it (A.D.G.); 3 IRCCS Ospedale Galeazzi Sant’Ambrogio, 20157 Milan, Italy; riccardo.giorgino@grupposandonato.it (R.G.); barbara.canciani@grupposandonato.it (B.C.); 4 Department of Precision and Regenerative Medicine and Ionian Area (DiMePreJ), University of Bari Aldo Moro, 70121 Bari, Italy

**Keywords:** osteoarthritis, adipose tissue, dog, mesenchymal cells, regenerative medicine

## Abstract

Osteoarthritis (OA) causes chronic pain and impaired mobility in dogs. Since current therapies cannot restore damaged articular cartilage, tissue engineering approaches offer promising therapeutic strategies. This study aimed to investigate whether peri-ovarian adipose tissue (POAT) represents a biologically competent and functionally relevant alternative source of mesenchymal stromal cells (MSCs) compared to subcutaneous adipose tissue (SAT). Samples were collected from five healthy and normal-weight Labrador Retrievers undergoing routine ovariectomy. MSCs were characterized according to the International Society for Cellular Therapy, including doubling time, growth curves, colony-forming unit assays, immunophenotyping, and trilineage differentiation potential. Chondrogenic differentiation was assessed through Alcian Blue staining and qPCR analysis of *COL2A1*, *COL1A1*, *COL10A1*, and *SOX9* expression at multiple timepoints. MSCs derived from both adipose depots showed comparable mesenchymal characteristics, proliferative capacity, immunophenotypic profiles, and multilineage differentiation potential. POAT-MSCs exhibited enhanced chondrogenic differentiation compared to SAT-MSCs, with stronger extracellular matrix deposition and significantly increased *COL2A1* expression at later stages of differentiation than SAT-MSCs. *SOX9* expression supported a more advanced chondrogenic commitment in POAT-derived cells, while *COL10A1* expression remained low and stable in both groups. These preliminary findings suggest that POAT, routinely discarded after ovariectomy, may represent a promising and ethically advantageous source of canine MSCs for regenerative medicine.

## Simple Summary

Osteoarthritis causes chronic pain and limits movement in dogs. Since current treatments cannot repair damaged joint cartilage, regenerative medicine offers new hope using stem cells. This study shows that the fat surrounding a female dog’s ovaries, a tissue normally thrown away after routine spay surgery, is an excellent and ethical source of cells with therapeutic and regenerative potential. Fat from five healthy Labrador Retrievers was collected, and mesenchymal stromal cells were isolated. Peri-Ovarian Adipose Tissue-derived cells showed a greater chondrogenic potential and produced a stronger cartilage-like extracellular matrix compared with subcutaneous adipose tissue-derived cells. These findings highlight the potential of peri-ovarian adipose tissue as a valuable resource for veterinary regenerative medicine, transforming surgical waste into a promising tool for future cartilage repair strategies in dogs affected by osteoarthritis.

## 1. Introduction

Articular cartilage is a specialized tissue composed of chondrocytes, embedded in an extracellular matrix rich in water, collagen, proteoglycans, and glycoproteins [1]. Due to its poor regenerative capacity and healing ability, cartilage can undergo irreversible, progressive alterations that can lead to osteoarthritis (OA). OA is a multifactorial, degenerative disease affecting synovial joints in both veterinary and human medicine [2,3]. Although several risk factors, such as age, sex, and excess body weight, are well established, the precise mechanisms that trigger the onset of the disease remain largely unclear, hindering early diagnosis and timely therapeutic intervention. As OA progresses, patients experience increasing pain and inflammation, along with a disruption of the normal balance between matrix synthesis and degradation, ultimately leading to the irreversible breakdown of articular cartilage [4].

In dogs, OA is a highly prevalent chronic condition (2.5–6.6%). It is frequently secondary to developmental or acquired orthopaedic diseases, including hip dysplasia, elbow dysplasia, patellar luxation, and cranial cruciate ligament (CCL) rupture [5].

Labrador Retrievers are among the breeds at highest risk for joint disorders such as CCL rupture and elbow dysplasia. They are also predisposed to obesity, a condition that further aggravates joint degeneration and functional impairment [6]. This predisposition may be partially explained by a deletion in the pro-opiomelanocortin gene, which has been associated with increased appetite, adiposity, and body weight in this breed [7].

Regenerative medicine offers innovative strategies for the repair of damaged joint cartilage, as demonstrated by numerous research studies and clinical trials investigating the role of mesenchymal stem cells (MSCs) in the treatment of OA [8]. In particular, under appropriate chondrogenic stimulation, MSCs can differentiate into chondrocyte-like cells and produce extracellular matrix components such as collagen, glycosaminoglycans (GAGs), and proteoglycans [9].

Adipose tissue is a well-established and highly accessible source of MSCs, particularly subcutaneous adipose tissue (SAT), which is widely used in canine regenerative studies [10,11]. These cells are defined according to International Society for Cellular Therapy (ISCT) criteria as plastic-adherent cells expressing mesenchymal markers, lacking hematopoietic markers, and exhibiting trilineage differentiation potential [12,13].

Increasing evidence indicates that MSC properties vary significantly depending on the anatomical origin of the adipose depot, including differences in proliferation rate, immunomodulation, and differentiation potential [14].

Previous studies demonstrated that canine ovarian and gonadal-associated tissues contain mesenchymal-like stromal cells with high proliferative activity and broad differentiation potential, suggesting that peri-gonadal regions may represent biologically active stem cell niches for regenerative applications, characterized by distinct MSC biological properties [14,15]. Despite increasing evidence of adipose depot-specific differences in MSC biology, limited information is currently available regarding the chondrogenic differentiation potential of peri-ovarian adipose tissue (POAT)-derived MSCs in homogeneous and clinically relevant canine models predisposed to obesity-associated orthopaedic disease [15,16].

Obesity is now recognized as a key factor impairing MSC biology, leading to reduced proliferative capacity, altered immunomodulation, and impaired differentiation potential, including chondrogenesis, in humans and companion animals. This effect is particularly relevant in dogs, where obesity is highly prevalent and strongly associated with orthopaedic disease progression. In this context, adipose-derived MSCs from obese or metabolically altered donors exhibit reduced regenerative efficiency and altered lineage commitment capacity [17,18,19,20]. Since the biological properties of MSCs are strongly influenced by the anatomical and metabolic characteristics of their tissue of origin, increasing attention has been directed toward understanding the functional heterogeneity among adipose depots and identifying novel MSC niches with distinct biological properties [21,22]. Among these, the POAT, associated with the ovarian bursa, is a gonadal-associated visceral fat depot exposed to a unique hormonal and metabolic microenvironment [23,24,25]. While obesity is well known to induce systemic low-grade inflammation and severely impair the regenerative potential of SAT-derived MSCs [17], we hypothesized that the specialized, hormone-buffered microenvironment of POAT might partially shield its resident stem cell niche from obesity-induced lipotoxic dysfunction. Indeed, local ovarian steroids, such as 17β-estradiol, have been widely demonstrated to exert potent anti-inflammatory, antioxidant, and anti-senescence protective effects on adipose-derived MSCs, preserving their survival and functionality [26]. However, no studies have directly investigated whether POAT-derived MSCs retain superior regenerative properties compared with SAT-derived MSCs under obese conditions. Rather, the biological distinctiveness of this depot warrants further investigation as a potentially relevant MSC source.

Unlike SAT, visceral fat is highly metabolically active, endocrine-responsive, and sensitive to systemic metabolic alterations such as obesity [27]. Notably, POAT is routinely discarded during elective ovariectomy procedures, making it a readily available, minimally invasive, and ethically advantageous MSC source compared to conventional SAT harvesting.

Accordingly, this study aimed to investigate whether POAT represents a biologically competent and functionally relevant alternative source of MSCs compared to SAT in a homogeneous cohort of young, healthy, normal-weight female Labrador Retriever. MSCs derived from both adipose depots were isolated from the same individuals and compared in terms of phenotype and multipotent differentiation capacity. In addition, chondrogenic differentiation potential was evaluated under in vitro induction conditions, to assess potential depot-related differences relevant to cartilage regeneration and canine orthopaedic disorders, which are highly prevalent in this species.

## 2. Materials and Methods

### 2.1. Experimental Design

SAT and POAT samples were collected from the same individuals, consisting of five clinically healthy female Labrador Retrievers undergoing elective ovariectomy. The animals were 10–14 months old and met the inclusion criteria of a normal body condition score (BCS 4–5/9), assessed according to the 9-point Laflamme scale [28,29]. The two adipose depots were therefore isolated from matched donors and directly compared. POAT was collected from the ovarian bursa during routine ovariectomy, whereas SAT was obtained from the linea alba during abdominal closure of the same surgical procedure. Ovariectomies were performed at the Lodi Veterinary Teaching Hospital (Department of Veterinary Medicine and Animal Sciences, University of Milan, Lodi), and samples were collected after informed consent from the owners. All experimental procedures were approved by the Institutional Ethics Committee (OPBA_137_2021).

SAT and POAT samples were divided into two portions for: I) morphological characterization of the tissue, and II) extraction and characterization of MSCs. Chondrogenic differentiation was initiated at the best-defined passage obtained in step II, and four experimental time points (T0, T7, T10, and T14 days in culture) were characterized at both genetic and morphological levels.

### 2.2. Morphological Characterization of the Tissue

After collection, samples were immediately fixed in 10% (*v*/*v*) neutral buffered formalin (Bio-Optica, Milan, Italy) overnight, rinsed under tap water and dehydrated through a graded ethanol series (Sigma-Aldrich, Saint Louis, MO, USA): 70% overnight at +4 °C, 95% for 1 h at room temperature (RT), and 100% for two 1-h steps at RT.

Subsequently, they were cleared in xylene (Sigma-Aldrich, Saint Louis, MO, USA) with two 1-h exchanges at room temperature (RT) and subsequently embedded in paraffin wax (Bio-Optica, Milano, Italy). Sections of 5 µm-thick were stained with haematoxylin and eosin (H&E) to assess tissue morphology. For each sample, 100 cells were independently analysed by two blinded operators to determine adipocyte area using Optika Pro View (version x64, 4.11.18081.20201205). The percentage of Stromal Vascular Fraction (SVF) was calculated as the ratio of the SVF area to the total image area, based on 10 images per sample, using ImageJ software (1.54g). Each animal was treated as an experimental unit, and statistical analyses were conducted using the mean value of all measurements taken per animal.

### 2.3. Cell Culture

Tissues intended for MSC extraction were collected under sterile conditions and kept in phosphate-buffered saline (PBS, ECM4053XL, EuroClone, Pero (MI), Italy) until processing in the cell culture laboratory. The tissues were weighed before processing to estimate cell yield per gram of tissue, then mechanically disintegrated using sterile forceps and scissors, and digested enzymatically in a shaking incubator at 38.5 °C in High-Glucose Dulbecco’s Modified Eagle’s Medium (HG-DMEM, ECB7501L, EuroClone, Italy) supplemented with 1% penicillin (100 IU/mL) and streptomycin (100 μg/mL) (P/S, A8948,0100 PanReac AppliChem, Italy), 0.25 μg/mL amphotericin B (Am, ECM009D, EuroClone, Italy), 1 mg/mL collagenase type I (C1-22-14, Sigma-aldrich, St. Louis, MO, USA), and 5 mM calcium chloride (CaCl_2_, C5080, Sigma-Aldrich, USA). Incubation continued for approximately 2 h, or until the tissue appeared completely disintegrated and ready for subsequent cell isolation steps. The digested suspension was filtered through an 80 μm cell strainer and centrifuged twice for 10 min at RT at 400× *g*. The resulting cell pellet was resuspended in an MSC culture medium consisting of HG-DMEM supplemented with 10% fetal bovine serum (FBS, ECS5000L, EuroClone, Italy), 10 ng/mL epidermal growth factor (EGF; PHG0315, ThermoFisher, Life Technologies Italia, Monza (MB), Italy), 1% P/S, 0.25 μg/mL Am, and 2 mM L-glutamine (Glu, ECB300D, EuroClone, Italy), as described by Lange-Consiglio et al. [30]. Viable cell counts were performed using the Trypan Blue (1450013, Bio-Rad Laboratories S.r.l., Segrate (MI), Italy) exclusion method on an automated cell counter (TC20 #1450102, Bio-Rad Laboratories S.r.l., Segrate (MI), Italy). Cells were seeded at a density of 100,000 cells/cm^2^, expanded to passage 1 (P1) at ~80% confluence, detached with 0.05% trypsin-EDTA (15400-054, Gibco, Life Technologies Italia, Segrate (MI), Italy) and frozen until use.

#### 2.3.1. Cell Proliferation Assays

Doubling time (DT) was assessed from P1 to passage 6 (P6) to identify the optimal passage balancing cell yield with the time required for culture expansion. At P1, 5000 cells/cm^2^ were seeded into a single well of a 6-well plate. Upon reaching confluence, cells were detached, counted, and reseeded at the same density into a new well at the next passage (P2). This process was repeated for each passage, with cells tracked from P1 through P6. For each sample, the DT was calculated using the standard formula: DT = (t2 − t1) × log (2)/log (N2/N1), where N1 is the cell number at the initial time point t1, and N2 is the cell number at the subsequent time point t2.

Once the optimal passages for use, as determined by DT, were identified, cell proliferation growth curves were monitored for 11 days in culture at P1, P2, and P3. In each condition, 5000 cells/cm^2^ were plated per well in a 6-well plate. Time zero (T0) was defined as 48 h after seeding. Subsequently, cells were counted at days 3, 5, 7, 9, and 11. For each time point, viability and cell number were recorded to generate the growth curves.

#### 2.3.2. Colony-Forming Unit (CFU) Assays

POAT-MSCs and SAT-MSCs were seeded and cultured for 7 days at P2 at three densities (25, 50, and 75 cells/cm^2^) to perform Colony-Forming Unit (CFU) assays. They were then fixed in formalin, stained with Crystal Violet (C3886, Sigma-Aldrich, USA), and colonies were counted as described by Lange et al. [30].

#### 2.3.3. Differentiation Assays

Chondrogenic, osteogenic, and adipogenic differentiation protocols were performed following established procedures. Cells were seeded at a density of 5000 cells/cm^2^ in a 6- well plate. For each lineage, different media were used. Chondrogenic differentiation medium consisted of HG-DMEM supplemented with 10% FBS, 1% penicillin–streptomycin–glutamine (P/S/G, 10378-016, Gibco, USA), 1% Am, 250 µg/mL L-ascorbic acid (AA, A8960-5G, Sigma-Aldrich, USA), 1% sodium pyruvate (ECM0542D, EuroClone, Italy), 100 µg/mL dexamethasone (D2915-100MG, Sigma-Aldrich, USA), 1% insulin–transferrin–selenium (ITS-A, Gibco), 1 ng/mL basic fibroblast growth factor (bFGF/FGF-2, 130093841, Miltenyi Biotec S.r.l., Bologna (BO), Italy), and 1 ng/mL transforming growth factor beta 3 (TGF-β3, 130-094-008, Miltenyi Biotec, Italy). Osteogenic differentiation medium consisted of HG-DMEM supplemented with 10% FBS, 1% P/S/G, 1% Am, 250 µg/mL L-AA, 10 µg/mL β-glycerophosphate (β-GP, G9422-10G, Sigma-Aldrich, USA), 100 µg/mL dexamethasone, and 1 ng/mL bFGF. Adipogenic differentiation was induced using an induction medium composed of HG-DMEM supplemented with 10% FBS,1% P/S/G, 1% AmB (Euroclone), 10 µg/mL insulin (I2643, Sigma-Aldrich, USA), 0.1 mM indomethacin (I7378-5G, Sigma-Aldrich, USA), 1 µM dexamethasone, 0.5 mM 3-isobutyl-1-methylxanthine (I5879-1G, Sigma-Aldrich, USA), and 1 ng/mL bFGF. Adipogenic maintenance medium consisted of HG-DMEM supplemented with 10% FBS, 1% P/S/G (Gibco), 1% AmB, 10 µg/mL insulin, and 1 µg/mL recombinant EGF. Cells were maintained in culture for seven days, with medium changes performed every two days. For adipogenic differentiation, an induction medium and a maintenance medium were alternated at each medium change to promote and sustain lineage commitment. On day seven, the cells were washed with PBS and fixed in 10% buffered formalin for 30 min. They were then immediately subjected to lineage-specific staining using Alcian Blue (Bio-Optica, Milan, Italy) for chondrogenic matrix deposition, Alizarin Red (Sigma-Aldrich, Saint Louis, MO, USA) for mineralization in osteogenic cultures, and Oil Red (Thermo Fischer Scientific, Erlenbachweg, Germany) for intracellular lipid accumulation in adipogenic cultures. After staining, the samples were imaged with an Axiophot microscope (Zeiss, Baden, Wurttemberg, Germany) equipped with a Leica DFC450 C camera (Leica Microsystems, Wetzlar, Germany).

#### 2.3.4. Flow Cytometric Immunophenotyping

Immunophenotypic characterization of the cells was performed via flow cytometry (FC) at P2. To assess the purity of the cell population, a comprehensive panel of surface markers was analysed, with a multicolour approach. Specifically, cells were screened for the expression of the mesenchymal associated markers (CD44 [IM7, FITC, Bio-Rad], CD90 [YKIX337.217, APC, ThermoFisher Scientific, Waltham, MA, USA], CD105 [polyclonal, PE, Bioss, Woburn (MA), USA), while hematopoietic and endothelial markers (CD11b [M1/70, PE-Cy5, ThermoFisher Scientific], CD31 [polyclonal, Purified (FITC-labelled secondary antibody), Biorbyt (Beckton Dickinson, San Josè (CA), USA)], CD34 [1H6, FITC, Bio-Rad], CD45 [YKIX716.13, AlexFluor647, Bio-Rad], and CD146 [P1H12, PE, ThermoFisher Scientific], along with the major histocompatibility complex marker MHCII, were used for negative selection. Staining procedures were performed according to already published protocols [31]. All samples were acquired with a bricyte E6 flow cytometer (Mindray, Shenzen, China) with constant settings and compensation matrix and analyzed with the dedicated software MrFlow (Mindray, version 01.11.00.8977) by a single experienced operator (VM). The percentage of antibody-positive cells out of total nucleated cells was recorded for each marker.

### 2.4. Chondrogenic Differentiation Capacity

To evaluate and compare the chondrogenic capacity of the two sources, four experimental time points were established: T0 (defined as the day on which cells reached 80% confluence); T7 (7 days after T0); T10 (10 days after T0), and T14 (14 days after T0). Based on preliminary analyses, P2 was identified as the most suitable passage and was therefore used for all subsequent analyses. The cells were seeded into the six wells of a 6-well plate at a density of 5000 cells/cm^2^ and cultured in MSC growth medium until 80% confluence was achieved. At this stage, samples corresponding to T0 were collected, whereas the remaining wells were washed in PBS, and the culture medium was replaced with chondrogenic differentiation medium. Differentiation was maintained for 7, 10 and 14 days, and in all experimental groups, the medium was changed twice weekly. For each time point, 6 wells were prepared: three wells were allocated for qPCR analysis, while the remaining three were designated for Alcian Blue staining. Upon reaching 80% confluence, wells intended for morphological assessment were fixed with 10% buffered formalin and immediately stained with Alcian Blue to assess glycosaminoglycan deposition as an indicator of chondrogenic differentiation.

Lysis buffer was added to the remaining three wells, and RNA was extracted using an RNeasy mini kit (QIAGEN, Hilden, Germany) according to the manufacturer’s instructions. Complementary DNA (cDNA) was synthesized from 500 ng of RNA using QuantiTect Reverse Transcription Kit (QIAGEN, Hilden, Germany), which includes a DNAse step. An additional reaction without the reverse transcriptase was performed to confirm the complete genomic contaminant DNA removal. The quality of cDNA was verified by conventional qualitative PCR amplifying the housekeeping genes.

Real-time PCR was performed using a Bio-Rad iQ5 Real-Time PCR System and using the SYBR^®^ Green Supermix (Bio-Rad, CA, USA) as a fluorescent molecule. The primer sequences for the amplification of Collagen type I alpha 1 chain (*COL1A1*), Collagen type II alpha 1 chain (*COL2A1*), Collagen type 10 alpha 1 chain (*COL10A1*), and SRY-box 9 (*SOX9*) gene fragments, and the corresponding bibliographic references are reported in Table 1. Genes coding for Hypoxanthine phosphoribosyltransferase 1 (*HPRT*) and Ribosomal protein L13 (*RPL* 13) were used as reference (Table 1).

Reactions were performed in a final volume of 20 microL with different final primer concentrations: 200 nM for *COL10A1*, *HPRT* and *RPL13*; 250 nM for *COL1A1* and *COL2A1*; 350 nM for *SOX9*. Thermal cycling conditions were as follows: 95 °C 15 s, 56 °C 15 s, 72 °C 10 s, for *SOX9*, *HPRT*; 95 °C 15 s, 60 °C 15 s, 72 °C 20 s, for *COL10A1*; 95 °C 15 s, 58 °C 30 s, for *SOX9* and *COL2A1*. A melting curve was performed to assess the specificity of amplification. Relative gene expression levels were calculated using the comparative ΔΔCt method. Expression values for each sample were normalized against the two housekeeping genes (*RPL13* and *HPRT*) and compared to SAT and POAT T0 samples used as reference.

### 2.5. Statistical Analysis

Statistical analyses were performed using GraphPad Prism software (version 8.0.1). Data normality was evaluated using the Shapiro–Wilk test to determine the appropriate comparative approach between the POAT and SAT groups. Accordingly, statistical significance was assessed using either the unpaired Student’s *t*-test for parametric data or the Mann–Whitney U test for non-parametric data. These analyses were applied to morphological characterization, doubling time, and flow cytometry data. To evaluate the effect of time, a two-way ANOVA followed by appropriate post hoc multiple comparison tests was employed (for growth curves and PCR analysis). Group comparisons for CFU assays were performed using a one-way ANOVA. Data are expressed as mean ± standard error of the mean (S.E.M.). Differences were considered statistically significant at *p* < 0.05.

## 3. Results

### 3.1. Morphological Characterization of the Tissue

**Figure 1:**
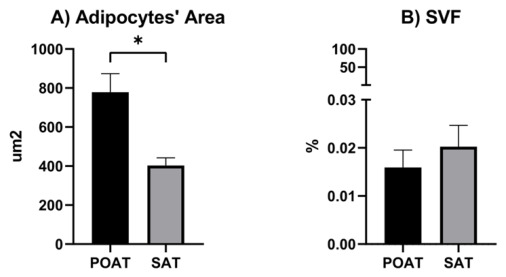
Morphological characterization of POAT and SAT. (**A**) Adipocytes’ area; (**B**) Stromal vascular fraction (SVF). Unpaired *t* test. N = 5 per experimental group. * = *p* < 0.5.

Adipocyte area were significantly higher in POAT than in the SAT (Figure 1A, *p* < 0.05). In contrast, there were no significant differences in stromal fraction between the two experimental groups (Figure 1B).

### 3.2. Isolation and Characterization of MSCs

#### 3.2.1. Cell Proliferation Assays

**Figure 2:**
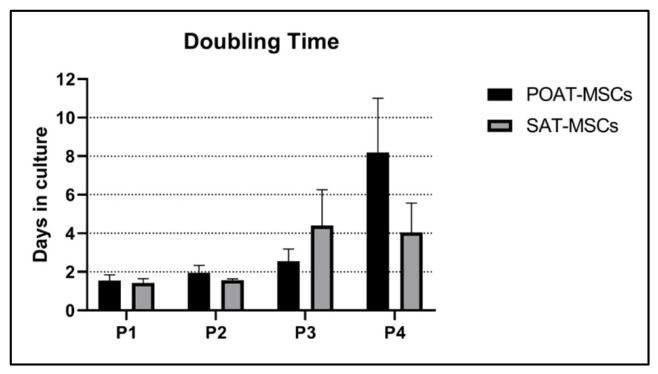
Doubling Time from passage 1 (P1) to passage 4 (P4). No statistically significant differences were observed between the experimental groups. Unpaired *t* test. N = 4 per experimental group.

Doubling times were determined from P1 to P4. Although P1 exhibited the lowest mean doubling time (mean ± S.E.M. for POAT-MSCs P1 = 1.548 ± 0.3012, P2 = 1.959 ± 0.3713, P3 = 2.560 ± 0.6259, P4 = 8.191 ± 2.811; mean ± S.E.M. for SAT-MSCs P1 = 1.429 ± 0.2149, P2 = 1.564 ± 0.07460, P3 = 4.400 ± 1.860, P4 = 4.043 ± 1.1524), P2 was considered the preferred passage for AT-MSC work due to the lowest inter-sample variability. From P3 onward, the coefficient of variation (C.V.) exceeded 50% between samples. No statistically significant differences were observed between the experimental groups (Figure 2).

From P5 to P6, the doubling time could not be determined because the cells failed to grow and instead died; the cell count remained below 50,000 cells per well. Consequently, DT could not be calculated.

**Figure 3:**
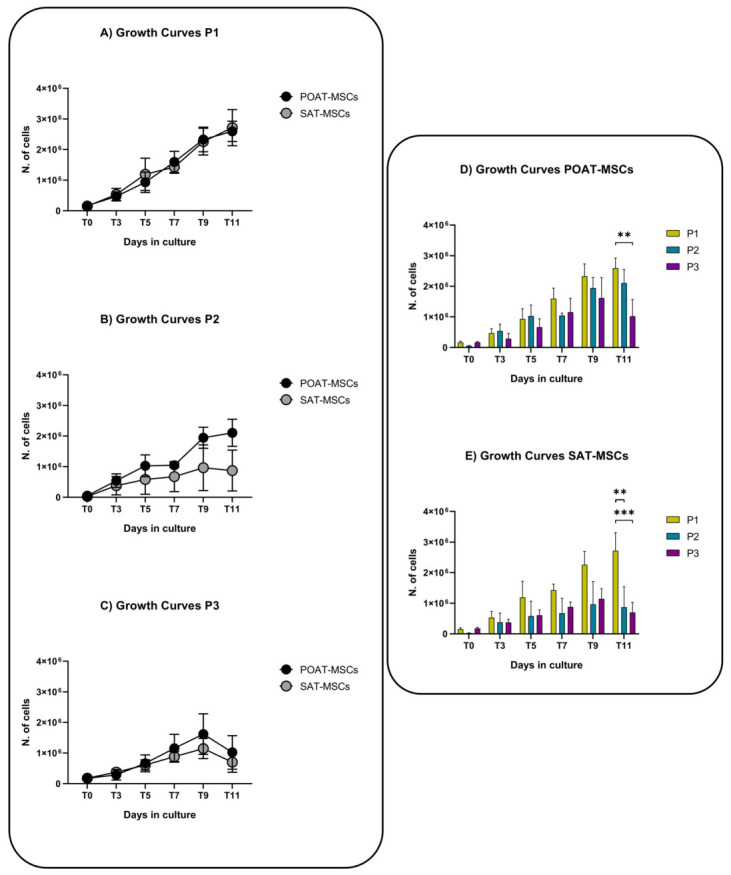
Growth curves of POAT-MSCs and SAT-MSCs at P1 (**A**), P2 (**B**), e P3 (**C**). Comparisons between passages are shown for POAT-MSCs (**D**) and SAT-MSCs (**E**). Statistical analysis was performed using two-way ANOVA. N = 3/4 per experimental group. ** = *p* < 0.01; *** = *p* < 0.001.

Regarding growth curves, no differences were observed between POAT-MSCs and SAT-MSCs at P1, P2, and P3 (Figure 3A–C). Similarly, no differences were observed up to T9 between P1, P2 and P3 in both POAT-MSCs and SAT-MSCs (Figure 3D,E), but after 11 days in culture, a significant reduction in the cell number was observed at P3 (Figure 3D,E) for both the types of cells.

#### 3.2.2. Colony-Forming Unit (CFU) Assays

**Figure 4:**
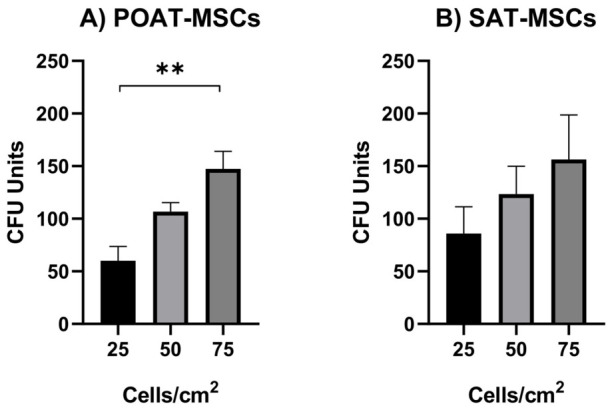
CFU frequency in POAT-MSCs (**A**) and SAT-MSCs (**B**). Data distribution was assessed using the Shapiro–Wilk normality test, followed by one-way ANOVA. N = 3 per experimental group. ** = *p* < 0.01.

Regarding CFUs, no differences were observed between cells derived from SAT and those derived from POAT. Nonetheless, POAT cells exhibited significant differences when seeded at 25 cells/cm^2^ compared with those seeded at 75 cells/cm^2^ (Figure 4A, *p* < 0.01).

#### 3.2.3. Differentiation Assays

**Figure 5:**
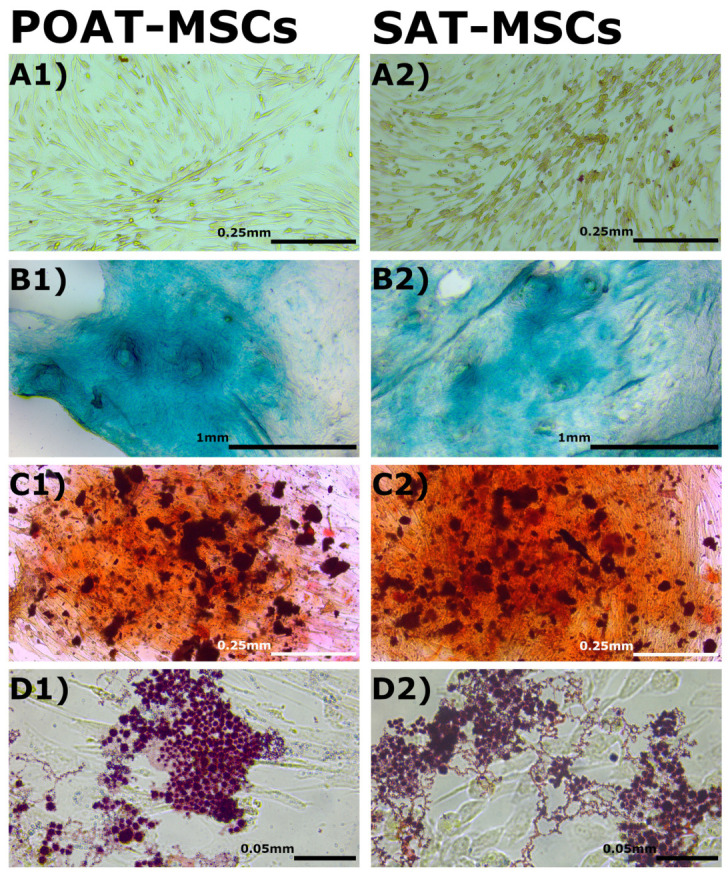
Multilineage differentiation potential of POAT-MSCs and SAT-MSCs. (**A1**–**D1**) POAT-MSCs and (**A2**–**D2**) SAT-MSCs after 7 days of induction. (**A1**,**A2**) Representative images of undifferentiated cells (control). Multipotency was assessed by (**B1**,**B2**) Alcian Blue staining for chondrogenic evaluation, (**C1**,**C2**) Alizarin Red S staining for osteogenic mineralization, and (**D1**,**D2**) Oil Red O staining for adipogenic lipid accumulation.

All three differentiations were completed within 7 days under the described seeding conditions. The cells showed a robust ability to adhere to the culture plate and rapidly acquired a characteristic fibroblast-like morphology. This morphology remained stable throughout the 7-day culture period, as shown in Figure 5(A1,A2) for cells maintained exclusively in control (undifferentiated medium). Cells induced toward chondrogenic differentiation were positive for Alcian Blue staining, indicative of cartilaginous matrix glycosaminoglycan production (Figure 5(B1,B2)). Similarly, cells cultured in osteogenic medium showed positive Alizarin Red staining consistent with calcium deposition (Figure 5(C1,C2)). Finally, cells subjected to adipogenic differentiation exhibited lipid accumulation, as confirmed by Oil Red O positivity (Figure 5(D1,D2)). In all experiments, negative controls (undifferentiated medium) were uniformly negative for all three histochemical stains (representative images in Figure 5(A1,A2)).

#### 3.2.4. Flow Cytometry

**Figure 6:**
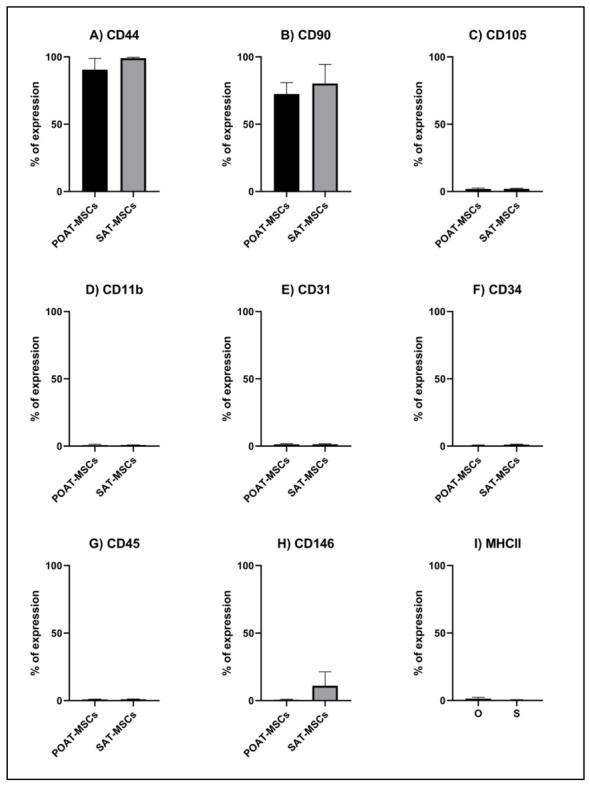
Immunophenotypic characterization of POAT-MSCs and SAT-MSCs via flow cytometry. Representative histograms showing that cells from both sources are strongly positive for mesenchymal markers CD44 (**A**) and CD90 (**B**). The mesenchymal marker CD105 was consistently negative in canine MSCs from both tissues (**C**). Both cell types were negative for all hematopoietic lineage markers (**D**–**H**), and for MHCII (**I**). Data distribution was assessed using the Shapiro–Wilk normality test; inter-group comparisons were performed using the unpaired *t*-test or Mann–Whitney U test, as appropriate. N = 3–4 per experimental group.

FC analysis showed that the cells were positive for the mesenchymal markers CD44 (Figure 6A) and CD90 (Figure 6B), but negative for CD105, which is commonly used for mesenchymal stem cell identification in other species (Figure 6C). All hematopoietic lineage markers (Figure 6D–H) and the MHC II marker (Figure 6I) were negative in all samples. No statistically significant differences were observed in the immunophenotypic profile between the two mesenchymal stem cell populations.

### 3.3. Chondrogenic Differentiation Capacity

**Figure 7:**
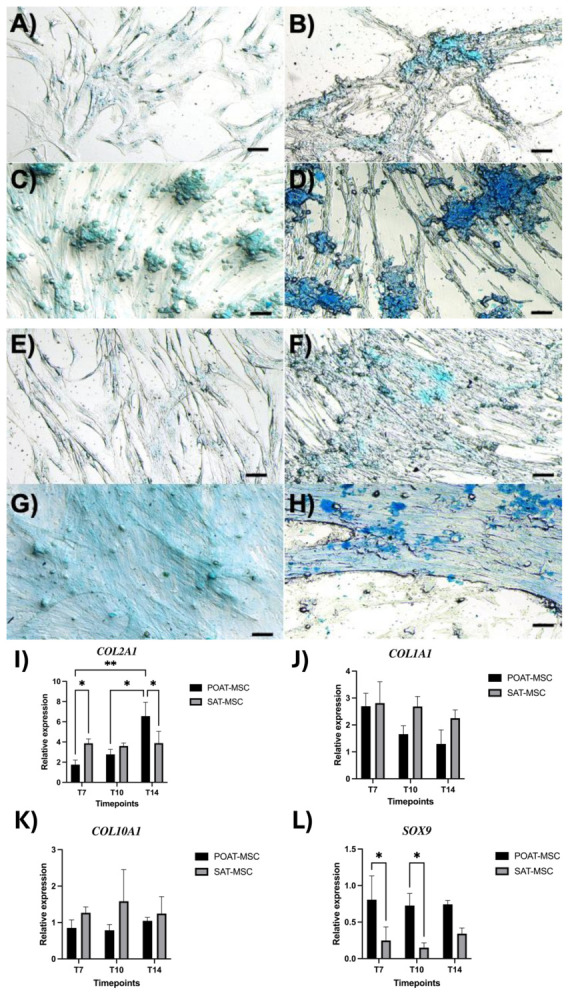
(**A**–**D**): Representative images of Alcian Blue staining of POAT-MSCs during chondrogenic differentiation. (**A**) T0: cells exhibit light blue staining mainly localized in the peri-nuclear area; (**B**) T7: Evident intracytoplasmic deposition of GAGs; (**C**) T10: Cells begin to lose their chondrogenic differentiation capacity, and Alcian Blue staining appears as small droplets; (**D**) T14: Marked aggregation of both cells and GAGs-rich droplets is observed. Scale bar 50 µm. (**E**–**H**): Representative images of Alcian Blue staining of SAT-MSCs during chondrogenic differentiation. (**E**) T0: cells show light blue staining in the peri-nuclear area; (**F**) T7: intracytoplasmic deposition of GAGs; (**G**) T10: limited extracellular deposition of GAGs; (**H**) T14: Limited deposition of GAGs and monolayer morphology. Scale bar 50 µm. (**I**–**L**): Gene expression of both POAT and SAT-MSCs. (**I**) *COL2A1* gene expression, T7 vs. T14: *p* < 0.01; T10 vs. T14: *p* < 0.05; (**J**) *COL1A1* gene expression; (**K**) *COL10A1* gene expression; (**L**) *SOX9* gene expression, T7 vs. T10: *p* < 0.05. * = *p* < 0.05; ** = *p* < 0.01.

At T0, Alcian Blue staining revealed a faint bluish tint in the peri-nuclear area of both tissue-derived cell types. By day 7, initial signs of ECM deposition became detectable in the cytoplasmic regions, indicating the onset of chondrogenic activity in both sources. By day 10, chondrogenic differentiation became evident in both POAT-MSCs (Figure 7A–D) and SAT-MSCs (Figure 7E–H). POAT-MSC-derived cells started forming multicellular aggregates and progressively lost their monolayer organization. Specifically, marked aggregation and GAGs-rich droplets were observed in the extracellular environment.

In contrast, SAT-MSC-derived cells largely preserved their monolayer architecture at day 10. Alcian Blue staining in these cultures was weaker and more diffuse, suggesting reduced glycosaminoglycan deposition compared to POAT-MSCs. By day 14, the features observed at day 10 were further accentuated in POAT-MSCs, with more pronounced matrix-rich aggregate formation. Meanwhile, SAT-MSCs, at this later stage, began to show signs of monolayer disruption, while still exhibiting limited matrix deposition compared to POAT-MSCs.

Regarding gene-expression analysis, no significant differences were detected between the experimental groups for *COL1A1* and *COL10A1* (Figure 7J,K). Notably, Coll 1 showed a decreasing trend in POAT, while remain stable in SAT-MSCs (Figure 7J). In contrast, qPCR revealed a higher *COL2A1* expression in POAT-MSCs (T7 vs. T14: *p* < 0.01; T10 vs. T14: *p* < 0.05), whereas in SAT-MSCs *COL2A1* expression remained stable over time. When comparing the two sources, POAT-MSCs exhibited lower *COL2A1* levels at T7 (*p* < 0.05) but significantly higher expression at T14 (*p* < 0.05) relative to SAT-MSCs. As shown in Figure 7L, *SOX9* expression was higher in POAT-MSCs compared to SAT-MSCs at T7 and T10 (*p* < 0.05). No statistically significant differences were detected in *COL10A1* expression (Figure 7K). Figure 7K shows that its levels remained low and stable in both POAT- and SAT-MSCs.

## 4. Discussion

This study provides a comparative characterization of mesenchymal stromal cells (MSCs) isolated from peri-ovarian adipose tissue (POAT) and subcutaneous adipose tissue (SAT) in young, healthy, normal-weight female Labrador Retrievers. The primary aim was to investigate whether POAT, a tissue routinely discarded during elective ovariectomy, may represent a biologically competent and clinically relevant alternative MSC source for regenerative medicine applications, particularly in the context of cartilage repair and canine osteoarthritis (OA).

Adipose tissue is currently considered one of the most accessible and clinically relevant MSC sources in both veterinary and human medicine due to the abundance of stromal cells, minimally invasive collection procedures, and high proliferative potential [10,11]. Nevertheless, increasing evidence indicates that MSC biological properties are strongly influenced by the anatomical origin of the adipose depot, including differences in proliferation, immunomodulation, secretory profile, and lineage differentiation potential [14]. In this context, the characterization of alternative adipose depots has become increasingly relevant, particularly for orthopedic diseases such as OA, in which cartilage regeneration remains a major therapeutic challenge.

Histological analyses performed in the present study revealed structural differences between the two adipose depots. POAT showed significantly larger adipocytes compared with SAT, whereas the stromal vascular fraction did not significantly differ between groups. These findings support the concept that adipose tissue heterogeneity extends beyond anatomical localization and involves intrinsic metabolic and structural characteristics of each depot. Similar depot-specific morphological differences have already been described in both humans and companion animals, particularly between visceral and subcutaneous adipose tissues, which exhibit distinct endocrine and immunometabolic profiles [27]. Since adipocyte size and tissue architecture are closely associated with metabolic activity and local cytokine production, these differences may partially contribute to the distinct biological behavior observed in the derived MSC populations.

To minimize methodological variability, both tissues were collected from the same donors and processed using identical surgical harvesting, enzymatic digestion, and culture protocols. This represents an important aspect of the experimental design, as previous studies demonstrated that MSC yield and biological performance may vary considerably depending on tissue handling and isolation procedures [34,35]. By standardizing all technical procedures, the observed differences are more likely attributable to intrinsic depot-specific biological properties rather than to procedural artifacts.

Both POAT- and SAT-derived cells exhibited classical mesenchymal characteristics, including plastic adherence, fibroblast-like morphology, and trilineage differentiation potential toward adipogenic, osteogenic, and chondrogenic lineages. In addition, both cell populations expressed the mesenchymal markers CD44 and CD90 while lacking hematopoietic and endothelial markers, confirming the mesenchymal identity of the isolated cells. Interestingly, CD105 expression was absent in both groups. Although CD105 is commonly included among the minimal criteria established by the International Society for Cellular Therapy (ISCT) for human MSC characterization [36], several studies reported variable or absent CD105 expression in canine MSCs, suggesting species-specific differences or culture-condition-dependent variability [37,38,39]. Therefore, our findings further support the notion that canonical human MSC immunophenotypic criteria may not be fully transferable to veterinary species.

Regarding proliferative properties, both MSC populations showed similar growth kinetics and doubling times under identical culture conditions. In both groups, proliferation was characterized by an early expansion phase followed by progressive reduction in proliferative efficiency from passage 3 onward, ultimately leading to cellular senescence at later passages. These findings are consistent with previous reports describing progressive senescence and reduced proliferative stability in canine MSCs after prolonged in vitro expansion [40]. Although passage 1 exhibited the lowest doubling times, passage 2 was selected for subsequent analyses because of its lower inter-sample variability and improved experimental reproducibility. Indeed, from passage 3 onward, the coefficient of variation between samples exceeded 50%, accompanied by a stark increase in the standard error of the mean. This escalation in sample heterogeneity at P3 and P4 reflects a well-documented challenge in primary MSC biology, driven by the intertwined effects of donor-to-donor variability and asynchronous replicative senescence [41,42,43]. While early passages capture the cells during a period of post-isolation stabilization, extended in vitro expansion amplifies the intrinsic biological differences, such as age or metabolic status, of individual donors. As the cell cycle decelerates non-uniformly across different donor lines, the statistical dispersion inevitably widens, fully justifying the selection of P2 as the optimal, standardized window for downstream experimental manipulations prior to the onset of senescence-induced batch effects. Notably, no significant differences in doubling time or growth curves were observed between POAT-MSCs and SAT-MSCs, indicating that the enhanced chondrogenic potential observed in POAT is unlikely to be merely related to differences in proliferative activity.

Similarly, CFU assays demonstrated comparable clonogenic capacity between the two MSC populations, although POAT-derived cells exhibited significantly increased colony formation at higher seeding densities. This finding may suggest a greater sensitivity of POAT-MSCs to paracrine signaling and cell–cell communication mechanisms, which are increasingly recognized as important regulators of MSC survival, self-renewal, and regenerative activity [44,45]. Although the biological significance of this observation remains speculative, it may reflect intrinsic differences in microenvironmental responsiveness between the two adipose depots.

The most relevant finding of this study concerns the enhanced chondrogenic differentiation potential observed in POAT-derived MSCs compared with SAT-derived cells. Cartilage regeneration remains one of the major limitations in OA treatment because mature articular cartilage exhibits limited intrinsic repair capacity due to its avascular nature and low cellular turnover [1]. Consequently, MSC-based regenerative strategies have attracted considerable attention in both veterinary and translational medicine, particularly for orthopedic disorders in dogs, including elbow dysplasia, cranial cruciate ligament disease, and OA [8].

Morphological evaluation through Alcian Blue staining demonstrated progressive extracellular matrix (ECM) deposition in both groups during chondrogenic induction. However, POAT-derived cells exhibited earlier and more pronounced formation of glycosaminoglycan-rich aggregates, together with progressive disruption of monolayer organization and development of multicellular structures typically associated with chondrogenic commitment. In contrast, SAT-derived cells maintained a more preserved monolayer morphology and showed weaker and more diffuse Alcian Blue positivity throughout differentiation. These observations suggest a more efficient acquisition of a chondrogenic phenotype in POAT-derived MSCs.

The molecular analyses further supported these morphological findings. *COL2A1* expression progressively increased in POAT-derived MSCs, reaching significantly higher levels at later differentiation stages compared with SAT-derived cells. Collagen type II represents one of the principal components of hyaline cartilage ECM and is widely recognized as a hallmark of mature chondrogenic differentiation. Therefore, the increased *COL2A1* expression observed in POAT-derived cells strongly supports a more advanced chondrogenic commitment. In contrast, SAT-derived MSCs showed relatively stable and lower *COL2A1* expression throughout differentiation, suggesting slower or less efficient progression toward a cartilage-like phenotype. Similar observations have been reported in previous studies, in which canine SAT-derived MSCs required longer induction periods to achieve robust *COL2A1* expression [46].

Interestingly, *COL1A1* expression displayed opposite trends in the two populations. While SAT-derived MSCs maintained relatively stable *COL1A1* levels, POAT-derived cells showed a progressive reduction over time. Increased *COL1A1* expression is generally associated with fibrocartilage or fibrotic tissue formation rather than hyaline cartilage differentiation [47]. Consequently, the concomitant increase in *COL2A1* and reduction in *COL1A1* observed in POAT-MSCs may indicate a more favorable progression toward a hyaline cartilage-like phenotype. This aspect is particularly relevant in cartilage tissue engineering, where the generation of fibrocartilage rather than hyaline cartilage remains one of the major limitations of MSC-based approaches.

*SOX9* expression patterns also support the enhanced chondrogenic commitment of POAT-derived cells. *SOX9* is considered one of the earliest and most important transcription factors involved in chondrogenesis, regulating the expression of cartilage-specific ECM components, including *COL2A1* [48]. In the present study, POAT-MSCs exhibited significantly higher SOX9 expression at earlier differentiation stages compared with SAT-derived cells, followed by progressive reduction over time concomitant with increasing *COL2A1* expression. This temporal pattern is consistent with physiological chondrogenic progression, in which early *SOX9* activation precedes extracellular matrix maturation and collagen type II production. Similar dynamics have been described in previous studies investigating MSC chondrogenesis [49].

Importantly, *COL10A1* expression remained low and stable in both groups throughout differentiation. Collagen type X is commonly associated with hypertrophic chondrocyte differentiation and endochondral ossification. Excessive *COL10A1* expression during MSC chondrogenesis is generally considered undesirable because it may indicate progression toward hypertrophic or osteogenic phenotypes rather than stable articular cartilage formation. Therefore, the absence of significant *COL10A1* upregulation suggests that the chondrogenic differentiation observed in POAT-derived MSCs was not accompanied by evident hypertrophic commitment under the adopted experimental conditions.

The enhanced chondrogenic potential observed in POAT-derived MSCs may be related to the unique biological characteristics of POAT. Unlike SAT, POAT represents a gonadal-associated visceral adipose depot exposed to a distinct endocrine and metabolic microenvironment closely influenced by ovarian physiology [50]. Visceral adipose tissues are known to exhibit higher metabolic activity, endocrine responsiveness, and cytokine production compared with subcutaneous fat [27]. Furthermore, previous studies demonstrated that gonadal-associated adipose tissues contain mesenchymal-like stromal populations with broad differentiation potential and high proliferative activity [14,15]. Therefore, it is plausible that the ovarian-associated microenvironment contributes to maintaining MSC populations with enhanced chondrogenic responsiveness.

Intriguingly, this distinct functional heterogeneity between adipose depots can be traced back to their developmental ontogeny and unique anatomical niches. Subcutaneous fat develops primarily from the paraxial mesoderm, whereas visceral and gonadal fat pads, including the POAT, originate from the lateral plate mesoderm during embryogenesis [51,52]. Cells derived from the lateral plate mesoderm in the urogenital region are tightly linked to the coelomic epithelium, a structure known for its high plastic potential and mesenchymal lineage commitment [53]. During embryogenesis, this specialized epithelial lining operates as a highly dynamic reservoir of multipotent progenitors, which locally supply crucial stromal elements to developing visceral organs via epithelial-to-mesenchymal transition (EMT), as demonstrated in visceral systems [53]. In the specific context of gonadogenesis and the development of surrounding serosal membranes, cells originating from this Wilms’ tumor protein 1 (Wt1)-expressing coelomic lineage actively delaminate and migrate into the nascent tissue architecture. Crucially, recent evidence highlights that mesenchymal stem cells derived specifically from the lateral plate mesoderm possess an intrinsically robust osteochondrogenic differentiation potential [54]. This specific embryonic track grants resident progenitors a profound developmental plasticity and a native propensity toward mesenchymal fates, effectively explaining the preserved, superior capacity of adult POAT-MSCs to undergo robust multi-lineage commitment, such as enhanced chondrogenesis, when compared to conventional subcutaneous depots. Furthermore, the periovarian niche exposes resident MSCs to a specialized microenvironment characterized by rich paracrine and endocrine signaling, including sex steroids and transforming growth factor-beta (TGF-β) superfamily members essential for follicular and gonadal homeostasis [23]. This continuous local exposure may epigenetically prime or pre-condition POAT-MSCs, resulting in an enhanced responsiveness to chondrogenic induction media compared to their subcutaneous counterparts.

Our results demonstrate that MSCs derived from both adipose depots satisfy the main mesenchymal criteria and exhibit comparable proliferative and multilineage differentiation properties. However, POAT-derived MSCs display enhanced chondrogenic commitment under in vitro induction conditions compared with SAT-derived cells, suggesting depot-related functional differences that may be relevant for cartilage regenerative strategies. From a translational perspective, POAT may represent a particularly attractive MSC source in veterinary medicine. This tissue is routinely discarded during elective ovariectomy procedures, making its collection minimally invasive, ethically advantageous, and potentially cost-effective. In addition, the possibility of obtaining biologically active MSCs during standard surgical sterilization procedures may facilitate future regenerative applications without requiring additional tissue harvesting interventions. This aspect may be especially relevant in dogs predisposed to orthopedic disorders, such as Labrador Retrievers, in which early regenerative strategies could potentially contribute to the management of OA progression.

Nevertheless, several limitations of this study should be acknowledged. First, the sample size was limited, and all donors consisted exclusively of young, healthy, normal-weight female Labrador Retrievers. Consequently, the findings cannot be directly generalized to other breeds, age groups, males, or obese animals. Since obesity and aging are known to significantly affect MSC biology and regenerative potential [55,56,57,58], further studies involving metabolically altered or osteoarthritic dogs will be necessary to validate the translational relevance of POAT-derived MSCs. Second, chondrogenic differentiation was evaluated exclusively using a conventional monolayer (2D) induction system rather than a high-density three-dimensional (3D) pellet culture model. While 3D pellet cultures represent the gold standard for replicating the physiological macromolecular organization and cell condensation required for cartilage-forming potential [36,59,60], monolayer induction serves as a highly standardized, reproducible platform for the preliminary comparative screening of chondrogenic differentiation capacity between distinct adipose depots [61,62]. Therefore, the findings of the present study should be interpreted as reflecting differences in chondrogenic differentiation capacity rather than definitive cartilage-forming ability. Consequently, additional investigations involving 3D biomimetic cultures or in vivo cartilage repair models are required to determine whether the enhanced chondrogenic commitment observed in vitro translates into improved cartilage regeneration in clinical settings. Finally, molecular analyses were limited to gene expression evaluation, and no protein-level validation or biomechanical characterization of the generated cartilage matrix was performed.

## 5. Conclusions

Despite these limitations, the present results provide preliminary evidence that POAT-derived MSCs possess enhanced chondrogenic differentiation potential compared with conventional SAT-derived MSCs while maintaining comparable mesenchymal and proliferative characteristics. These results support the concept that adipose depot origin significantly influences MSC biological behavior and suggest that peri-ovarian adipose tissue may represent a promising alternative MSC source for canine cartilage regenerative medicine and translational orthopedic research.

## Figures and Tables

**Table 1:** List of primers used in qPCR for each target. Pf: Primer Forward; Pr: Primer Reverse; Ref: references.

Target Gene	Pf	Pr	Ref.
*COL1A1*	GTGTGTACAGAACGGCCTCA	TCGCAGATCACGTCATCG	[32]
*COL2A1*	GCAGCAAGAGCAAGGAC	TTCTGAGAGCCCTCGGT	[32]
*COL10A1*	TTCCAGGACAGCCAGGCATCA	TTCCCAGTGCCTTCTGGTCC	[33]
*HPRT*	CACTGGGAAAACAATGCAGA	ACAAAGTCAGGTTTATAGCCAACA	[15]
*RPL13*	GGAGAAGGCCAGAGTCATCACA	TTTGCCCTGATGCCAAAAAG	[15]
*SOX9*	CGCTCGCAGTACGACTACAC	GGGGTTCATGTAGGTGAAGG	[32]

## Data Availability

The raw data supporting the conclusions of this article will be made available by the authors on request.

## Abbreviations

The following abbreviations are used in this manuscript:

OA	Osteoarthritis
SAT	Subcutaneous adipose tissue
POAT	Peri-ovarian adipose tissue
MSC	Mesenchymal stromal cells
CCL	Cranial cruciate ligament
GAGs	Glycosaminoglycans
ISCT	International Society for Cellular Therapy
PBS	Phosphate-buffered saline
HG-DMEM	High-Glucose Dulbecco’s Modified Eagle’s Medium
CaCl_2_	Calcium chloride
FBS	Fetal bovine serum
EGF	Epidermal growth factor
Glu	L-glutamine
DT	Doubling time
CFU	Colony-forming unit
P/S/G	Penicillin-streptomycin-glutamine
AA	L-ascorbic acid
ITS	Insulin-Transferrin-selenium
bFGF/FGF-2	Basic fibroblast growth factor
TGF-β3	Transforming growth factor beta 3
β-GP	β-glycerophosphate
FC	Flow cytometry
COL1A1	Collagen type I
COL2A1	Collagen type II
COL10A1	Collagen type 10
HPRT1	Hypoxanthine phosphoribosyltransferase 1
RPL13	Ribosomal protein L13
S.E.M.	Standard error of the mean

## References

[1] Sophia Fox A.J., Bedi A., Rodeo S.A. (2009). The basic science of articular cartilage: Structure, composition, and function. Sports Health.

[2] Sergio M., Karjalainen V.-P., Das Gupta S., Millar V.R.H., Mirra G., Di Giancamillo M., Modina S., Sconfienza L.M., Mangiavini L., Peretti G.M. (2025). Prolonged excessive weight induces spontaneous meniscal degeneration in sows: A preclinical model for obesity-related knee OA. Ann. Anat..

[3] Theyse L.F.H., Mazur E.M. (2024). Osteoarthritis, adipokines and the translational research potential in small animal patients. Front. Vet. Sci..

[4] Reihs E., Fischer A., Gerner I., Windhager R., Toegel S., Zaucke F., Rothbauer M., Jenner F. (2025). Beyond symptomatic alignment: Evaluating the integration of causal mechanisms in matching animal models with human pathotypes in osteoarthritis research. Arthritis Res. Ther..

[5] Pye C., Bruniges N., Peffers M., Comerford E. (2022). Advances in the pharmaceutical treatment options for canine osteoarthritis. J. Small Anim. Pract..

[6] Witsberger T.H., Villamil J.A., Schultz L.G., Hahn A.W., Cook J.L. (2008). Prevalence of and risk factors for hip dysplasia and cranial cruciate ligament deficiency in dogs. J. Am. Vet. Med. Assoc..

[7] Raffan E., Dennis R.J., O’dOnovan C.J., Becker J.M., Scott R.A., Smith S.P., Withers D.J., Wood C.J., Conci E., Clements D.N. (2016). A Deletion in the Canine POMC Gene Is Associated with Weight and Appetite in Obesity-Prone Labrador Retriever Dogs. Cell Metab..

[8] Canciani B., Rossi N., Arrigoni E., Giorgino R., Sergio M., Aidos L., Di Giancamillo M., Millar V.R.H., Peretti G.M., Di Giancamillo A. (2024). In Vitro Characterization of Human Cell Sources in Collagen Type I Gel Scaffold for Meniscus Tissue Engineering. Gels.

[9] Le H., Xu W., Zhuang X., Chang F., Wang Y., Ding J. (2020). Mesenchymal stem cells for cartilage regeneration. J. Tissue Eng..

[10] Choudhery M.S., Badowski M., Muise A., Pierce J., Harris D.T. (2015). Subcutaneous Adipose Tissue-Derived Stem Cell Utility Is Independent of Anatomical Harvest Site. BioRes. Open Access.

[11] Ritter A., Friemel A., Roth S., Kreis N.-N., Hoock S.C., Safdar B.K., Fischer K., Möllmann C., Solbach C., Louwen F. (2019). Subcutaneous and Visceral Adipose-Derived Mesenchymal Stem Cells: Commonality and Diversity. Cells.

[12] Czerwiec K., Zawrzykraj M., Deptuła M., Skoniecka A., Tymińska A., Zieliński J., Kosiński A., Pikuła M. (2023). Adipose-Derived Mesenchymal Stromal Cells in Basic Research and Clinical Applications. Int. J. Mol. Sci..

[13] Zhang J., Liu Y., Chen Y., Yuan L., Liu H., Wang J., Liu Q., Zhang Y. (2020). Adipose-Derived Stem Cells: Current Applications and Future Directions in the Regeneration of Multiple Tissues. Stem Cells Int..

[14] Rashid U., Yousaf A., Yaqoob M., Saba E., Moaeen-Ud-Din M., Waseem S., Becker S.K., Sponder G., Aschenbach J.R., Sandhu M.A. (2021). Characterization and differentiation potential of mesenchymal stem cells isolated from multiple canine adipose tissue sources. BMC Vet. Res..

[15] Trindade A.B., Therrien J., Garcia J.M., Smith L.C. (2017). Mesenchymal-like stem cells in canine ovary show high differentiation potential. Cell Prolif..

[16] Ishiuchi N., Nakashima A., Maeda S., Miura Y., Miyasako K., Sasaki K., Uchiki T., Sasaki A., Nagamatsu S., Nakao N. (2023). Comparison of therapeutic effects of mesenchymal stem cells derived from superficial and deep subcutaneous adipose tissues. Stem Cell Res. Ther..

[17] Oestreich A.K., Collins K.H., Harasymowicz N.S., Wu C.L., Guilak F. (2020). Is Obesity a Disease of Stem Cells?. Cell Stem Cell.

[18] Tvarijonaviciute A., Ceron J.J., Holden S.L., Cuthbertson D.J., Biourge V., Morris P.J., German A.J. (2012). Obesity-related metabolic dysfunction in dogs: A comparison with human metabolic syndrome. BMC Vet. Res..

[19] Xu H., Barnes G.T., Yang Q., Tan G., Yang D., Chou C.J., Sole J., Nichols A., Ross J.S., Tartaglia L.A. (2003). Chronic inflammation in fat plays a crucial role in the development of obesity-related insulin resistance. J. Clin. Investig..

[20] Kealy R.D., Lawler D.F., Ballam J.M., Mantz S.L., Biery D.N., Greeley E.H., Lust G., Segre M., Smith G.K., Stowe H.D. (2002). Effects of diet restriction on life span and age-related changes in dogs. J. Am. Vet. Med. Assoc..

[21] Ferreira-Baptista C., Ferreira R., Fernandes M.H., Gomes P.S., Colaço B. (2023). Influence of the Anatomical Site on Adipose Tissue-Derived Stromal Cells’ Biological Profile and Osteogenic Potential in Companion Animals. Vet. Sci..

[22] Farag A., Samir H., Ngeun S.K., Kaneda M., Hendawy H., Takahashi K., Tanaka R. (2025). Comparative cardiomyocyte differentiation potential of rat adipose-derived mesenchymal stem cells from two anatomical sites: Metabolomic profiling and pathway analysis. Front. Cell Dev. Biol..

[23] Szyrzisko W., Grzesiak M. (2024). Periovarian Adipose Tissue—An Impact on Ovarian Functions. Physiol. Res..

[24] Anaya E.S., de Groot E.L., Lydon J.P., Pangas S.A., Hartig S.M. (2024). Contributions of white adipose tissue to energy requirements for female reproduction. Trends Endocrinol. Metab..

[25] Ünal M.S., Uysal A., Gözlükaya T.S., Önder E., Seçme M., Çil N., Tabatabaei S., Mete G.A. (2025). Characterization and comparison of mesenchymal stem cells derived from rat perirenal and periovarian adipose tissue. Pamukkale Med. J..

[26] Mirzamohammadi S., Mehrabani M., Tekiyehmaroof N., Sharifi A.M. (2016). Protective effect of 17β-estradiol on serum deprivation-induced apoptosis and oxidative stress in bone marrow-derived mesenchymal stem cells. Hum. Exp. Toxicol..

[27] Ibrahim M.M. (2010). Subcutaneous and visceral adipose tissue: Structural and functional differences. Obes. Rev..

[28] Chun J.L., Bang H.T., Ji S.Y., Jeong J.Y., Kim M., Kim B., Lee S.D., Lee Y.K., Reddy K.E., Kim K.H. (2019). A simple method to evaluate body condition score to maintain the optimal body weight in dogs. J. Anim. Sci. Technol..

[29] Kipperman B.S., German A.J. (2018). The Responsibility of Veterinarians to Address Companion Animal Obesity. Animals.

[30] Lange-Consiglio A., Corradetti B., Bizzaro D., Magatti M., Ressel L., Tassan S., Parolini O., Cremonesi F. (2012). Characterization and potential applications of progenitor-like cells isolated from horse amniotic membrane. J. Tissue Eng. Regen. Med..

[31] Riondato F., Poggi A., Miniscalco B., Sini F., Marconato L., Martini V. (2023). Flow Cytometric Features of B- and T-Lmphocytes in Reactive Lymph Nodes Compared to Their Neoplastic Counterparts in Dogs. Vet. Sci..

[32] Teunissen M., Verseijden F., Riemers F.M., van Osch G.J.V.M., Tryfonidou M.A. (2021). The lower in vitro chondrogenic potential of canine adipose tissue-derived mesenchymal stromal cells (MSC) compared to bone marrow-derived MSC is not improved by BMP-2 or BMP-6. Vet. J..

[33] Endo K., Fujita N., Nakagawa T., Nishimura R. (2019). Comparison of the effect of growth factors on chondrogenesis of canine mesenchymal stem cells. J. Vet. Med. Sci..

[34] Schneider S., Unger M., Van Griensven M., Balmayor E.R. (2017). Adipose-derived mesenchymal stem cells from liposuction and resected fat are feasible sources for regenerative medicine. Eur. J. Med. Res..

[35] Kocan B., Maziarz A., Tabarkiewicz J., Ochiya T., Banaś-Ząbczyk A. (2017). Trophic Activity and Phenotype of Adipose Tissue-Derived Mesenchymal Stem Cells as a Background of Their Regenerative Potential. Stem Cells Int..

[36] Dominici M., Le Blanc K., Mueller I., Slaper-Cortenbach I., Marini F.C., Krause D.S., Deans R.J., Keating A., Prockop D.J., Horwitz E.M. (2006). Minimal criteria for defining multipotent mesenchymal stromal cells. The International Society for Cellular Therapy position statement. Cytotherapy.

[37] Yasumura Y., Teshima T., Nagashima T., Michishita M., Shigechika H., Taira Y., Suzuki R., Matsumoto H. (2025). Canine adipose-derived mesenchymal stromal cells inhibit the growth of canine hematologic malignancy cell lines. Regen. Ther..

[38] Yasumura Y., Teshima T., Michishita M., Suzuki R., Matsumoto H. (2025). A prospective single-arm study on the effects of repeated intravenous infusions of canine adipose-derived mesenchymal stromal cells in dogs with chronic inflammatory enteropathy. Stem Cell Res. Ther..

[39] Burdzinska A., Szopa I.M., Majchrzak-Kuligowska K., Roszczyk A., Zielniok K., Zep P., Dąbrowski F.A., Bhale T., Galanty M., Paczek L. (2024). The Comparison of Immunomodulatory Properties of Canine and Human Wharton Jelly-Derived Mesenchymal Stromal Cells. Int. J. Mol. Sci..

[40] Jankowski M., Dompe C., Sibiak R., Wąsiatycz G., Mozdziak P., Jaśkowski J.M., Antosik P., Kempisty B., Dyszkiewicz-Konwińska M. (2020). In Vitro Cultures of Adipose-Derived Stem Cells: An Overview of Methods, Molecular Analyses, and Clinical Applications. Cells.

[41] Ren J., Stroncek D.F., Zhao Y., Jin P., Castiello L., Civini S., Wang H., Feng J., Tran K., Kuznetsov S.A. (2013). Intra-subject Variability in Human Bone Marrow Stromal Cell (BMSC) Replicative Senescence: Molecular Changes Associated with BMSC Senescence. Stem Cell Res..

[42] Kannan S., Gokul Krishna S., Gupta P.K., Kolkundkar U.K. (2024). Advantages of pooling of human bone marrow-derived mesenchymal stromal cells from different donors versus single-donor MSCs. Sci. Rep..

[43] Quintero-Gil C., Jaraba-Álvarez W.V., Machuca-Acevedo C., Gómez V., Halpert K., Jiménez D., Ortega-Arellano H. (2026). Beyond Passage Numbers: How Culture Conditions and Population-Doubling Metrics Reporting Shape the Quality of Umbilical Cord-Derived MSCs and Extracellular Vesicles. Int. J. Mol. Sci..

[44] Peshkova M., Korneev A., Suleimanov S., Vlasova I.I., Svistunov A., Kosheleva N., Timashev P. (2023). MSCs’ conditioned media cytokine and growth factor profiles and their impact on macrophage polarization. Stem Cell Res. Ther..

[45] Kadir N.D., Yang Z., Hassan A., Denslin V., Lee E.H. (2021). Electrospun fibers enhanced the paracrine signaling of mesenchymal stem cells for cartilage regeneration. Stem Cell Res. Ther..

[46] Voga M., Drnovsek N., Novak S., Majdic G. (2019). Silk fibroin induces chondrogenic differentiation of canine adipose–derived multipotent mesenchymal stromal cells/mesenchymal stem cells. J. Tissue Eng..

[47] Mueller M.B., Tuan R.S. (2008). Functional characterization of hypertrophy in chondrogenesis of human mesenchymal stem cells. Arthritis Rheumatol..

[48] Bi W., Deng J.M., Zhang Z., Behringer R.R., De Crombrugghe B. (1999). *SOX9* is required for cartilage formation. Nat. Genet..

[49] Di Giancamillo A., Deponti D., Modina S., Tessaro I., Domeneghini C., Peretti G.M. (2017). Age-related modulation of angiogenesis-regulating factors in the swine meniscus. J. Cell. Mol. Med..

[50] Berryman D.E., List E.O., Sackmann-Sala L., Lubbers E., Munn R., Kopchick J.J. (2011). Growth hormone and adipose tissue: Beyond the adipocyte. Growth Horm. IGF Res..

[51] Billon N., Dani C. (2011). Developmental Origins of the Adipocyte Lineage: New Insights from Genetics and Genomics Studies. Stem Cell Rev. Rep..

[52] Chau Y.Y., Bandiera R., Serrels A., Martínez-Estrada O.M., Qing W., Lee M., Slight J., Thornburn A., Berry R., McHaffie S. (2014). Visceral and subcutaneous fat have different origins and evidence supports a mesothelial source. Nat. Cell Biol..

[53] Wilm B., Ipenberg A., Hastie N.D., Burch J.B.E., Bader D.M. (2005). The serosal mesothelium is a major source of smooth muscle cells of the gut vasculature. Development.

[54] Wei Y., Wang B., Jia L., Huang W., Xiang A.P., Fang C., Liang X., Li W. (2022). Lateral Mesoderm-Derived Mesenchymal Stem Cells With Robust Osteochondrogenic Potential and Hematopoiesis-Supporting Ability. Front. Mol. Biosci..

[55] Lee J., Lee K.S., Kim C.-L., Byeon J.S., Gu N.-Y., Cho I.-S., Cha S.-H. (2017). Effect of donor age on the proliferation and multipotency of canine adipose-derived mesenchymal stem cells. J. Vet. Sci..

[56] Taguchi T., Borjesson D.L., Osmond C., Griffon D.J. (2019). Influence of Donor’s Age on Immunomodulatory Properties of Canine Adipose Tissue-Derived Mesenchymal Stem Cells. Stem Cells Dev..

[57] Bertolo A., Steffen F., Malonzo-Marty C., Stoyanov J. (2015). Canine Mesenchymal Stem Cell Potential and the Importance of Dog Breed: Implication for Cell-Based Therapies. Cell Transplant..

[58] Heyman E., Olenic M., De Vlieghere E., De Smet S., Devriendt B., Thorrez L., De Schauwer C. (2025). Donor age and breed determine mesenchymal stromal cell characteristics. Stem Cell Res. Ther..

[59] Watts A.E., Ackerman-Yost J.C., Nixon A.J. (2013). A Comparison of Three-Dimensional Culture Systems to Evaluate In Vitro Chondrogenesis of Equine Bone Marrow-Derived Mesenchymal Stem Cells. Tissue Eng. Part A.

[60] Boeuf S., Richter W. (2010). Chondrogenesis of mesenchymal stem cells: Role of tissue source and inducing factors. Stem Cell Res. Ther..

[61] Ruhl T., Beier J.P. (2019). Quantification of chondrogenic differentiation in monolayer cultures of mesenchymal stromal cells. Anal. Biochem..

[62] Prosser A., Scotchford C., Roberts G., Grant D., Sottile V. (2019). Integrated Multi-Assay Culture Model for Stem Cell Chondrogenic Differentiation. Int. J. Mol. Sci..

